# The long-term effect of the Great Recession on European mortality

**DOI:** 10.1007/s12546-022-09290-8

**Published:** 2022-08-08

**Authors:** Giambattista Salinari, Federico Benassi

**Affiliations:** 1 Department of Economics and Business, University of Sassari, Sassari, Italy; 2 Italian National Institute of Statistics (ISTAT), Rome, Italy

**Keywords:** Procyclical mortality, Great Recession, Difference-in-differences, Life expectancy, Europe

## Abstract

Some European countries, such as Greece and Spain, were severely hit by the 2008 economic crisis whereas others, such as Germany, were practically spared by it. This divergence allowed us to implement a difference in differences research design which offered the possibility to observe the long-lasting effects produced by the crisis on European life expectancy. Our analysis—based on Eurostat data from 2001 to 2019—shows that life expectancy increased faster, after the onset of the crisis, in those countries where the rise in unemployment was more intense. Furthermore, our results show that this gain in life expectancy persisted, and sometimes further increased, until 2019 when most macro-economic variables had returned to their pre-crisis values. Previous research has identified that mortality behaves procyclically in developed countries: when the economy slows down mortality decreases and vice versa. Our findings show, by contrast, that life expectancy behaves asymmetrically: it responded to an increase but not to a decrease in unemployment. This calls for a reconsideration of the causal mechanisms linking together the economic cycle and mortality in developed countries.

## Introduction

Many analyses, conducted with different methods on aggregated data, have identified a counterintuitive *short-term* association between the economic cycle and mortality: when economic conditions deteriorate mortality tends to decrease, and vice versa. The, so called, *procyclical* behavior of mortality was first identified in the Unites States by Ogburn and Thomas (1922) (see Tapia Granados, 2015). The finding was then reconfirmed for the United States, in the 1970s, in the analyses conducted by Eyer (1977, 1985), and then again in the years 2000s in the works of Ruhm (2000, 2005, 2007, 2016), Tapia Granados (2005a, 2005b) and Stevens et al. (2015). Analyses conducted in other countries with advanced economies present similar results. In the case of Europe, for instance, mortality has been found to follow a procyclical dynamic in Germany (Neumayer, 2004), England and Wales (Tapia Granados, 2012), Spain (Tapia Granados, 2005c) and Norway (Haaland & Telle, 2015), but not in France (Brüning & Thuilliez, 2019). In Sweden results were mixed: Gerdtham and Johannesson (2005) using micro data find evidence of a countercyclical relationship (mortality declines when the economy improves), whereas Tapia Granados and Ionides (2008), using time series analysis, identify procyclical dynamics for the second half of the twentieth century. Outside Europe, a procyclical mortality dynamic was found in Japan (Tapia Granados, 2008), Canada (Ariizumi & Schirle, 2012), New Zealand (Greenaway-McGrevy, 2021) but not in Australia (Khemka & Roberts, 2015). Mortality was found to be procyclical in OECD countries by Gerdtham and Ruhm (2006).

The Great Recession that started in 2008 gave researchers the possibility to observe how mortality responds to an economic shock of extreme intensity (Toffolutti & Suhrcke, 2014; Tapia Granados & Ionides, 2017; Baumbach & Gulis, 2014; Ballester et al., 2019; Cervini-Plá & Vall-Castelló, 2021; Regidor et al., 2016). These analyses, mainly conducted in the European countries most affected by the crisis, give more support to the hypothesis of procyclical mortality dynamics.

Research works conducted on developing countries, instead, returned mixed results. Evidence of a counter cyclical dynamic was found by Bhalotra (2010) in India, by Sun et al. (2021) in China, and by Arroyave et al. (2015) in Colombia, whereas a procyclical dynamics was found in Mexico by Quast and Gonzalez (2014) and in the Asia–Pacific countries by Lin (2009).

The idea that in more developed countries mortality may follow procyclical dynamics has, of course, triggered a lively debate among specialists (see, for instance, the debate published in the International Journal of Epidemiology in 2005, Volume 34, Issue 6). The main problem brought up in relation to this hypothesis was that it contradicted several well-established findings from individual level analyses on the relationship between mortality, on the one hand, and unemployment, income, and social status, on the other. As pointed out by Catalano and Bellows (2005) and Catalano et al. (2011) in a systematic review of the issue, individual level analyses systematically show that unemployment is generally associated with a higher likelihood of depression, anxiety, cardiovascular diseases, premature death, and suicide. From this fact Catalano and Bellows conclude that the procyclical behavior of mortality identified by aggregated level analyses must be an ecological fallacy. In the analyses on the procyclical dynamic of mortality, however, it is now generally recognized that unemployment and poverty entail bad health consequences for individuals who are directly implicated. At the same time, it is claimed that the slowdown of the economy, proxied by the increase in unemployment, may entail some hidden beneficial effect for the overall population through different and complex pathways (Regidor et al., 2019).

First, it has been observed that when the economy slows down people tend to change lifestyle reducing smoking, drinking, excessive eating and increasing the time devoted to physical activity, sport, and medical care. This behavior has been explained by the lower time prices associated to the crisis and has been identified by individual level analyses (Catalano et al., 2011). Second, at aggregate level, health may be considered as an input for the economy so that a temporary economic decline may entail less air pollution, fewer work accidents, job related stress, longer patterns of sleep etc. all factors that may entail major effects on respiratory and cardiovascular diseases. Third, during economic crises commercial mobility and work interactions also decline provoking a reduction in the number of car accidents and in the deaths due to transmittable diseases. Actually, the analyses conducted on different subgroups of the population (by age, sex, income, social status, level of education) have shown that the procyclical dynamics of mortality seems to affect the entire population, simultaneously. Though different sub-groups are affected with different intensities (Regidor et al., 2016; Stevens et al., 2015).

Although different methods have been devised to investigate the connection between the economic cycle and mortality, by far the most common are time series analyses for a single geographic location and panel data analysis for several geographic locations at several points in time. More recently, panel analysis seems to be preferred to time series analysis in these kinds of studies. In panel data models, life expectancy at birth or age standardiszed mortality rates are generally employed as dependent variables. The key explanatory variable is represented by the rate of unemployment, which is preferred to other economic indicators such as, for instance, GDP *per capita*. This preference depends on unemployment being better able to capture the moment when an economic (financial) crisis spreads to the entire population. In a panel model, the association between mortality and unemployment is gauged by adding to the model location fixed effect, time fixed effect, location specific linear trend and other control variables (typically, demographic characteristics such as the share of the population over 65, or indicators of the level of education etc.) intended to reduce the possibly spurious associations between unemployment and mortality. Economou et al. (2008) have criticized this last point claiming that among the set of control variables lifestyle factors such as smoking, drinking, dietary habits, medical interventions, and the like should also be included. They show for nine European countries that once lifestyle factors are added to the model the connection between the economic cycle and mortality changes from the procyclical to the countercyclical. Although the addition to the model of lifestyle factors is worth debating, Economou’s et al (2008) work is important because it shows that the models employed in this kind of analyses are sensitive to the choice of covariates. As regards lifestyle factors, the problem consists in that they are probably not shared causes (confounders) of unemployment and mortality, and thus they are not expected to produce an omitted variable bias. These variables are usually viewed as intermediate variables (mediators), meaning that the slowdown of the economy is supposed to cause an improvement in health conditions exactly by inducing a modification in people’s lifestyle. Given their mediating nature, lifestyle factors represent what Angrist and Pischke (2009) call a “bad control” and should accordingly be excluded from the model (see on that also Pearl, 2009).

In the present paper we set out to analyse the *long-term* effect, over eleven years, of the Great Recession on European mortality. As we have seen, all explanations of the procyclical mortality dynamic insist on a short-term connection between economic cycles and mortality. It would thus be expected that, after the peak of the crisis, mortality should return to its pre-crisis dynamic. In this work we show that this is not the case for Europe. Using a difference-in-differences (DD) research design we show that, eleven years after the onset of the crisis, mortality still follows, in the countries most affected by the recession, a more favourable dynamic with respect to countries less affected by it. The DD research design employed in this study rests on a set of assumptions which are significantly different from those employed in time series or panel data analysis. From this point of view, the present study can be viewed as a robustness check on the well-established connection between the economic cycle and mortality. However, our findings also show that the economic crisis had a more long-lasting effect than expected, which calls for a reconsideration of the causal mechanisms linking together the economic cycle and mortality dynamics.

## Data and methods

Several papers have thoroughly analysed the short-term effects of the Great Recession in Europe supporting the idea of a procyclical behavior of mortality. One of these produced by Tapia Granados and Ionides (2017), focuses on 27 European countries: from the European Union only Luxembourg, Malta, and Cyprus are excluded because of their too small demographic size; there is also Norway, Switzerland, and the United Kingdom. These countries were classified into three main groups according to the severity of the effects produced by the crisis on the rate of unemployment.

- The *first group* comprises nine countries where the crisis had no or only mild effects (the variation in the rate of unemployment, $\Delta U$, was less than 2 percentage points between 2007 and 2010): Austria, Belgium, Finland, France, Germany, The Netherlands, Norway, Romania, and Switzerland.
- The *second group* consists instead of eleven countries which suffered a moderate crisis ($2<\Delta U\le 4$): Bulgaria, Croatia, the Czech Republic, Denmark, Hungary, Italy, Poland, Portugal, Slovakia, Sweden, and the UK.
- The *third group* includes seven countries which experienced severe effects $\Delta U>4$: Estonia, Greece, Ireland, Latvia, Lithuania, Slovenia, and Spain.

In the following we will use this classification to compare the evolution of mortality in the first *control group* (no or only mild effects) with that in the remaining countries, the *treated group*. Of course, economic crises are multifaceted phenomena, and it is probably impossible to fully capture their dynamics with a single proxy such as unemployment increase. For this reason, in the next two sections, we will perform several checks to assess the validity of the proposed classification of the European countries.

Period life expectancy at birth or at age 65 as provided by Eurostat for the period 2001–2019 has been employed as the mortality indicator. We preferred this indicator because it is not affected by the age structure of the population and because, as demonstrated by Lee (2019), Oeppen and Vaupel (2002) and White (2002), it tends to evolve over time following approximately a linear trend with a steady slope. This property of life expectancy will be useful in the application of the DD research design. We started our analysis in 2001, because data on life expectancy is not available in Eurostat for two countries, Croatia and Latvia, before this date. We decided, instead, to stop the analysis in 2019 to protect the analysis from the perturbing effects of the COVID-19 pandemics.

Among the countries here analysed, the recession—defined as a reduction in seasonally controlled real GDP over at least two consecutive quarters—appeared earlier in Italy (Q3-2007) and later in Belgium, Croatia, the Netherlands, Slovenia, Spain (Q4-2008) and the Czech-Republic (Q1-2009). The end of the recession ranges, instead, from Q2-2009 in Germany and Portugal to Q1-2010 in Spain and Q3-2010 in Croatia (see Ballester et al., 2019). It has been known for a long time that unemployment is a lagged indicator of the business cycle, so that it raises with a lag when the economy slows down and declines with a lag when the economy starts growing again (Mitchell, 1923). For this reason, we will consider in this work the year 2009 as year 0 of the crisis (this point will be further investigated in the next two sections).

We used Eurostat data to implement a DD research design (Angrist & Pishke, 2009; Cunningham, 2021; Hernán & Robins, 2020). This is the oldest among the causal inference methods. It was first used by John Snow in 1855 to show that cholera propagates through water, and it has since then been employed in uncountable contexts including epidemiology, economics, demography and political science. In very recent times it has been used to show that the Affordable Care Act which expanded Medicaid eligibility in the US produced a reduction in mortality in the states where it was present (Miller et al., 2021).

**Fig. 1:**
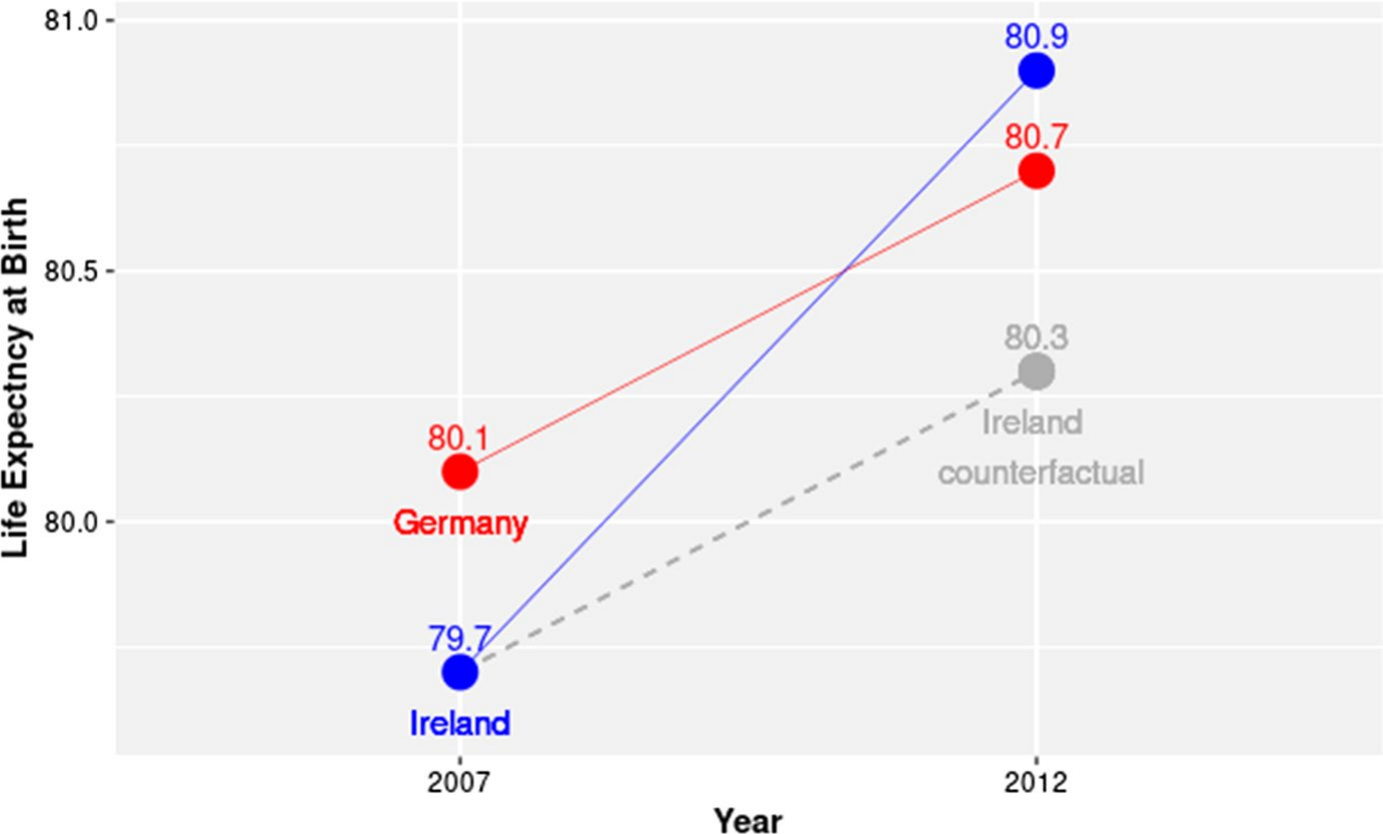
Example of a 2 × 2 difference-in-differences analysis. *Data*: Authors’ computation on Eurostat data

In its simplest form the DD research design requires two units (the control and the treated unit) and two different time points (before and after the treatment). Figure 1 gives an example of this simplified version of the DD research design. Here we consider two units, Germany and Ireland, for which we know that they were respectively marginally and severely affected by the crisis: Germany being the control unit and Ireland the treated unit. For these two countries we observe life expectancy in 2007, before the onset of the crisis, and in 2012 when unemployment was at its zenith in Europe.

According to the DD procedure the crisis produced in Ireland a gain in life expectancy of 0.6 years. This value is called the average effect of the treatment on the treated (ATT). The ATT was computed, in this case, by subtracting from the actual value (80.9) of life expectancy in Ireland in 2012, the potential (counterfactual) value of life expectancy (80.3) that would have been observed had the crisis not affected the Irish economy. The key aspect of the DD research design is the method through which the counterfactual value for Irish life expectancy in 2012 is calculated. This is done by assuming that the evolution of Irish life expectancy would have followed the same evolution as German life expectancy had the crisis not been there. This is the so-called *parallel trends assumption*. On top of that, the DD research design also assumes that future treatment does not affect past outcomes: in other terms, units do not change their behaviour before treatment, because they know they will be treated.

The parallel trends assumption is certainly a strong assumption, but not stronger than those commonly used in more “standard” analyses. In panel data analysis, for instance, it is assumed that all backdoor (confounding) paths have been blocked by the inclusion into the model of a proper set of time-varying covariates (the adjustment set) specifically aimed at preventing an omitted variable bias. However, in most analyses of this kind, variables such as unemployment (the treatment) and mortality (the outcome) can be thought to be simultaneously influenced by numerous common causes: for instance, the proportion of people with no diploma; proportion of migrants; proportion of the labor force employed in agriculture or in industry. Given this complexity, most of these analyses can only hope to reduce the entity of the omitted variable bias, but not to eliminate it. For this reason, it is considered good research practice to complement these analyses with others based on a different set of assumptions.

The DD research design offers probably the most popular alternative to standard methods. Furthermore, the parallel trends assumption appears particularly well suited in the analysis of mortality insofar as countries with similar characteristics are considered. This is because it has been shown that in Western countries, the dynamics of life expectancy has followed an approximately linear evolution (Lee, 2019; Oeppen & Vaupel, 2002) around a steady slope of 3 months of gain in life expectancy every year.

Given the parallel trends assumption the ATT can be formally computed in a 2 × 2 DD design as:

1 $${\text{ATT }} = \, \left( {{\text{Treated}}\,{\text{Post }}{-}{\text{ Treated}}\,{\text{Pre}}} \right) \, {-} \, \left( {{\text{Control}}\,{\text{Post }}{-}{\text{ Control}}\,{\text{Pre}}} \right)$$

where Treated Post and Treated Pre indicate, respectively, the post-crisis (≥ 2009) and the pre-crisis (< 2009) value of life expectancy in the country affected by the crisis, and Control Post and Control Pre represent the post- and pre-crisis life expectancy in the country not affected by the crisis.

In a linear regression setting, the ATT can be estimated through the following model:

2 $$Y_{c,t} = \beta_{c} + \gamma_{t} + \delta D_{c,t} + \varepsilon_{c,t}$$

where $Y_{c,t}$ is life expectancy in country *c* at time *t*, the $\beta_{c}$ are country fixed effects, the $\gamma_{t}$ are time fixed effects, $D$ is a binary variable assuming value 1 if the country went into a moderate or severe crisis and the country is observed after the crisis onset, and 0 otherwise, and $\varepsilon$ is the error term. In the case of the example shown in Fig. 1, the estimation of Eq. (2) through OLS resolves in the estimation of a saturated model. This notwithstanding it can be shown that the $\delta$ parameter is an estimate of the ATT (Angrist & Pischke, 2009; Cunningham, 2021). It should be noted, in this regard, that we cannot exclude the possibility that at least some of the countries in our control group suffered a mild form of recession. We will, therefore, consider in the present context the estimates of the $\delta$ coefficient as a lower bound for the ATT: we will consider, accordingly, that the gain in life expectancy observed in Ireland was *at least* 0.6 years.

Equation (2) can be employed to analyse situations characteriszed by multiple units (countries) and periods (years). However, Bertand et al. (2004) have shown that if multiple time periods are used the standard error computed in the usual way may result in a downward biased with the consequence that the null hypothesis of a zero $\delta$ coefficient is over rejected. For this reason, they suggest employing clustered standard errors at the country level. Clustering allows for an arbitrary serial correlation and heteroskedasticity at the country level over time. When estimated over the entire period covered by our data (2001–2019) the $\delta$ coefficient captures the gain in life expectancy observed in the treated group after the onset of the crisis (2009–2019) compared to the control group. However, in the present context we want also to know the effect of the crisis at specific points in time to assess whether the crisis entailed a short-term or a long-term effect on life expectancy (or neither). To this end, we estimated Eq. (2) focusing, first, on the years 2008 and 2012, and then on the years 2008 and 2019. By considering only the years 2008 and 2012 we gauge the effect of the crisis when unemployment is at its peak in Europe; by considering the years 2008 and 2019 we measure, instead, the effect of the crisis when the unemployment rate has returned to its pre-crisis level. The last two analyses differ from that covering the whole period 2001–2019 in an important way. When the DD analysis spans the years 2001–2019 it is assumed that the parallel trends hypothesis holds for this entire period. By contrast, when the analysis is restricted to one pre- and one post-crisis year, the parallel trends assumption is supposed to hold only for these two specific years, not for the entire period.

Although the key assumption of parallel trends cannot ultimately be tested, since no one can know for sure what would have been the value of Irish mortality in 2012 had the crisis not affected the Irish economy, two methods have been devised to investigate the plausibility of such a hypothesis. The first check is represented by the so-called “placebo test”. In the original version of this procedure, Eq. (2) is applied to two different groups of untreated units, in which one of them is (fictitiously) considered as treated. In this kind of analysis, we thus expect that the estimated $\delta$ coefficient turns out not significantly different from 0. If, contrary to expectation, the estimated $\delta$ coefficient proves to be significantly different from zero, this will identify a potential problem in the DD procedure. Since we do not have a second group of untreated units to hand, we modified the logic of the placebo test by splitting our data into two different sub-data sets. The first sub-data set includes the countries of group 1 (no or mild crisis) and those of group 2 (moderate crisis); the second data set includes, again, the countries of group 1 (no or mild crisis) and those of group 3 (severe crisis). We then estimated the coefficients of Eq. (2) separately in these two sub-data sets. In this modified version of the placebo test, we thus expect the $\delta$ coefficient estimated in the second sub-data set to be larger than that estimated in the first one, because a larger increase in unemployment should be associated to larger gains in life expectancy. If this is not the case, then the check will have identified a potential problem in the DD procedure. Notice that, in the present context, the proposed placebo test can also be viewed as a check on the goodness of the classification of the European countries used in this study.

The second check that we perform is probably the most important for our purposes. This time the analysis covers the entire period over which our data extend, from 2001 to 2019. The year when the intervention takes place (2009) is set as time 0, $t \in \left\{ { - 8, - 7, \cdots , 10} \right\}$. The check focuses on the period before the intervention to test if a trend of the same type as that identified after the intervention can be detected before it as well. In the present context, this verifies whether the countries affected by the crisis show a faster increase in life expectancy even before its onset. If this happens, this will indicate, of course, a bad DD design. To perform this test the following equation is estimated:

3 $$Y_{c,t} = \beta_{c} + \gamma_{t} + D_{c} \mathop \sum \limits_{t \ne - 1} \delta_{t} + \varepsilon_{c,t}$$

where $D$ is a binary variable indicating whether a given country went into a moderate or severe crisis (belongs to group 2 or 3) and the $\delta_{t}$ coefficients represent the deviation in the evolution of life expectancy observed between the treated and the control group. Note that the year before the crisis has been set as reference category in the model (we follow here Miller et al., 2021). If the $\delta_{t}$ coefficients are not significantly different from the reference value before the crisis (*t* < 0) and then make a jump at the onset of the crisis (*t* ≥ 0) this will prove the robustness of the DD procedure.

In the present context we will use Eq. (3) beyond its usual purpose of checking the robustness of the DD procedure (we follow in that, again, Miller et al., 2021). In the next section (“Unemployment changes during and after the Great Recession”) we will use the estimates of the ${\delta }_{t}$ coefficients to assess the effect of the economic crisis on unemployment in Europe (to this end we will use as dependent variable the unemployment rates provided by the World Bank). This will allow us to check the timing of the onset of the crisis and the goodness of the classification proposed by Tapia Granados and Ionides (2017). In the fourth section, devoted to the analysis of the effect of the crisis on life expectancy, we will use the estimates of the ${\delta }_{t}$ coefficients to assess the evolution of life expectancy in the 11 years (from 2009 to 2019) after the crisis. If the crisis entails a short-term effect on life expectancy we should see, after time − 1, the ${\delta }_{t}$ coefficients first increasing and then declining, reflecting the evolution of the unemployment rate.

## Unemployment changes during and after the Great Recession

The Great Recession first arrived in Europe in the last quarter of 2007 and then spread across the whole continent in the two following years. The Eastern and the Mediterranean European countries were comparatively more affected by the crisis, whereas Central and Western Europe were relatively less impacted. This was, in fact, the second global recession of the second millennium, the first one being the one that occurred during the years 2000–2001.

In this section we want to analyse the effect of the Great Recession on the unemployment rate (defined as a percentage of the total labor force) in the 27 European countries covered by the analysis. More specifically, we want here to address two important issues for our next analysis of life expectancy. The first issue concerns the timing of the crisis. We are, in this regard, particularly interested in understanding when the unemployment rate started to increase and whether the onset of the crisis was simultaneous in all European countries. The second issue concerns, instead, the goodness of the classification of the European countries according to the severity of the effects produced by the crisis (Tapia Granados & Ionides, 2017). Since we are going to use this classification in the analysis of life expectancy, we need first to verify its reliability.

**Fig. 2:**
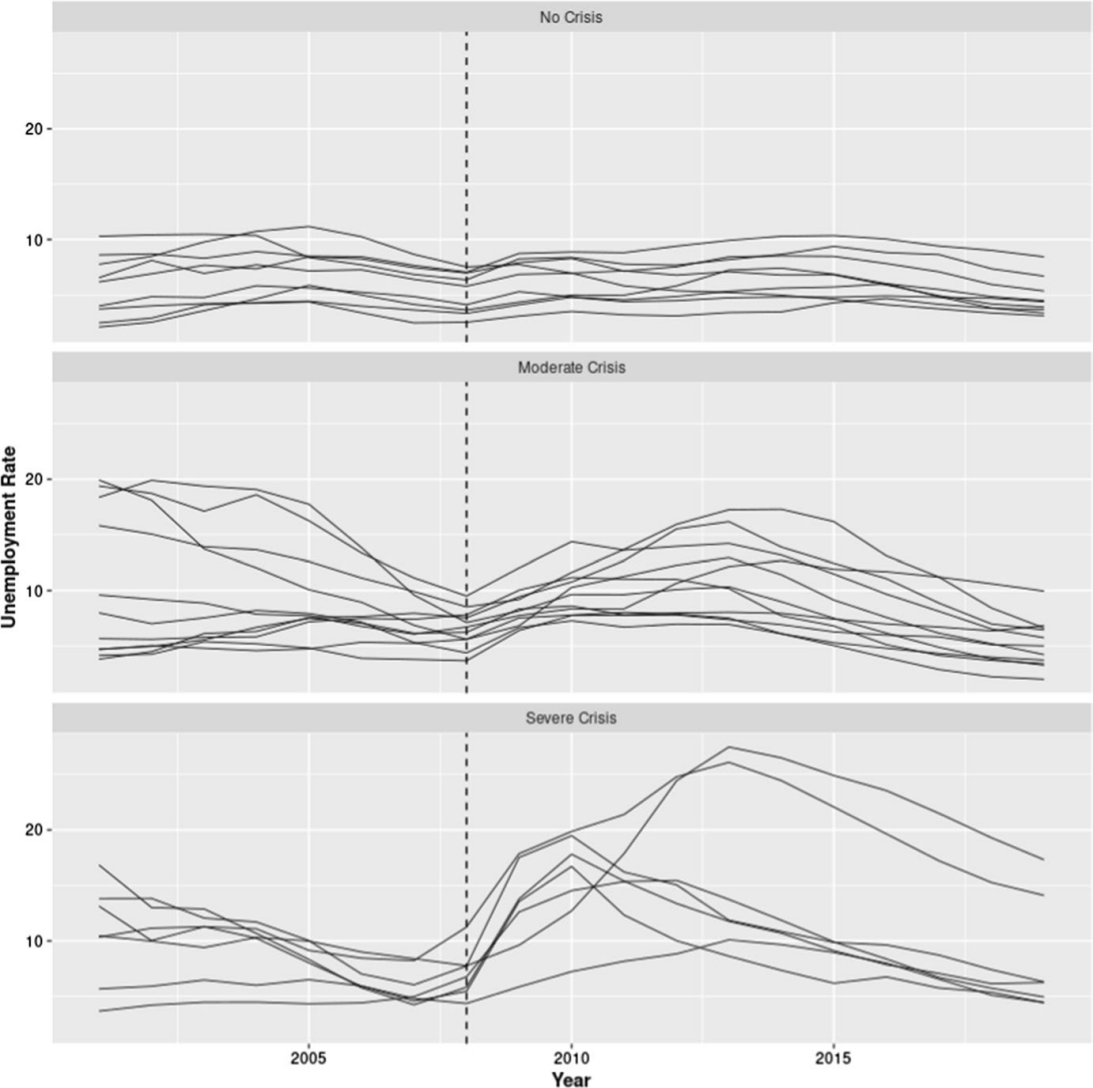
Evolution of the unemployment rate (% of total labor force) in 27 European countries. *Note:* Countries have been classified into three main groups (no, moderate, and severe crisis) following the classification proposed by Tapia Granados and Ionides (2017)*.* The vertical dashed line indicates the last pre-crisis year, 2008. *Source*: World Bank

With these aims in mind we have represented, in Fig. 2, the evolution of the unemployment rate over the period 2001–2019 for each of the 27 European countries. These countries have been classified according to the severity of the effect produced by the crisis in three groups: (1) No (or mild) crisis; (2) Moderate crisis; (3) Severe crisis (see “Data and methods” section for further details). The vertical dashed lines in the three panels of Fig. 2 indicate the year 2008.

Figure 2 shows that in most European countries the years before 2009 were characteriszed by a decline in the unemployment rate. This probably represents the end of the recovery phase following the 2000–2001 crisis. Actually, in most of the countries the year 2008 was the year in which the unemployment rate was at its lowest level. Consistently with what has been already identified for previous crises, the increase in the unemployment rate follows the financial crisis with a certain lag, so for most of the countries in our data set a significant rise in unemployment can be observed only starting from 2009. The group of countries more severely affected by the crisis represents a partial exception to this timing, with some countries of this group showing a slight rise in unemployment already in 2008. It must be said, however, that even for these countries the most dramatic increase in unemployment appears to take place in 2009. We then opted to identify the year 2009 as the onset (t = 0) of the crisis.

The data displayed in Fig. 2 also appears supportive of the classification proposed by Tapia Granados and Ionides (2017). The countries classified as only slightly affected by the crisis show only minor variations in the rate of unemployment over the entire period 2001–2019. The countries affected by a moderate form of recession, instead, experienced from 2009 a notable increase in unemployment which in most of the countries reached a peak in 2012. In the third group two countries, Greece, and Spain, stand out for their high unemployment levels.

In 2013 this value exceeded in both countries the staggering value of 25%. In the remaining countries of this group the crisis had less dramatic consequences with a level of unemployment oscillating in the peak year around 15%.

It should be noted that the crisis appears to have unfolded on a slightly different timetable in the countries of the second (moderate crisis) and of the third group (severe crisis). While in some countries in these two groups the crisis had a very fast evolution with a peak in unemployment already in 2010 followed by a long period of recovery, in others the crisis had more lasting effects with the peak in unemployment occurring two or three years later. In any case, after the years 2012–2013 all European countries analysed here show a decline in the unemployment rate. This is a key point for our analysis because it is in this last period that we expect to see disappearing a reduction of the beneficial, but supposedly temporary, effect for health produced by the crisis.

**Fig. 3:**
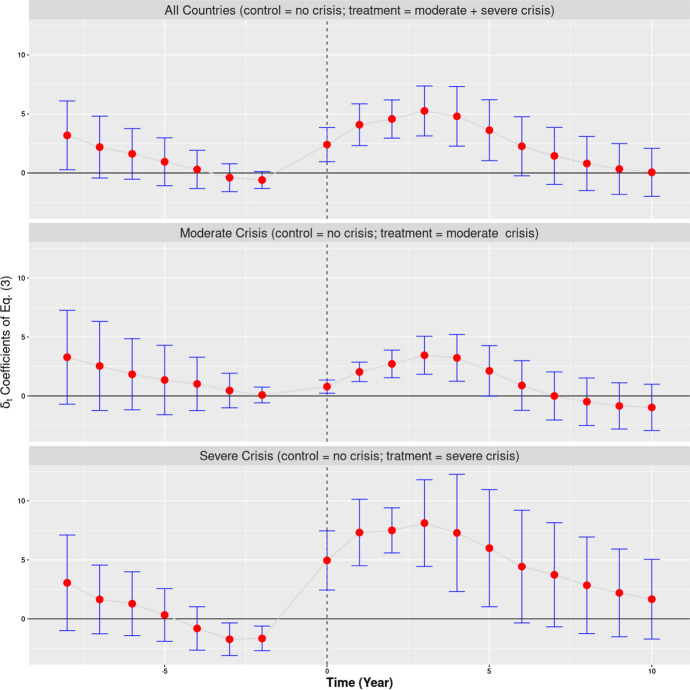
Changes in unemployment by level of severity of the Great Recession. *Note:* This figure represents the estimated $\delta_{t}$ coefficients of Eq. (3) along with their 95% confidence intervals. The dependent variable is, in this case, the unemployment rate. In the three analyses displayed by this figure (All Countries, Moderate Crisis and Severe Crisis) the control group is always represented by the countries that suffered no crisis or a mild form of it. In the first panel (*All Countries*) the model has been estimated for all 27 countries taken together, considering as treated group the countries that suffered a moderate or a severe crisis. In the second panel (*Moderate Crisis*, 20 countries) the treated group is represented by the countries that went through a moderate crisis, whereas the countries that experienced a severe crisis were dropped from the analysis. In the third panel (*Severe Crisis*, 16 countries), the treated group is represented by the countries that went through a severe crisis, whereas the countries that experienced a moderate crisis were dropped from the analysis. The vertical dashed line indicates the first crisis year, 2009. *Source*: Authors’ computation on World Bank data

Figure 3 displays the results of a more analytic investigation of the diffusion of the crisis in Europe. On the vertical axis, we have displayed the estimates of the ${\delta }_{t}$ coefficients of Eq. (3). These values represent the estimated deviation of the treated group from the evolution of the control group expressed in terms of the last pre-crisis year (the reference). In all three analyses displayed in Fig. 3, the control group is always represented by the group of countries that did not experience the crisis. The three analyses differ, instead, in the choice of the treated group. In the first panel (all countries) the treated group is represented by group 2 (moderate crisis) and 3 (severe crisis) taken together. In the second panel, the treated group is represented by group 2 alone. In the third panel, the treated group is represented by group 3 alone. The first year of the crisis (2009, t = 0) is marked in Fig. 3 by a vertical dashed line.

Figure 3 shows that, overall, there was not a significant difference between the control and the treated group in the 3–4 pre-crisis years, once the time invariant characteristics of the countries are adjusted for. Actually, if we consider as the treated group the set of countries that experienced a severe form of crisis, the three to four years leading up to the crisis appears to be somewhat more favourable, in terms of unemployment level, compared to the control group. The arrival of the crisis radically changed this situation: whatever the (treated) group of countries considered, the ${\delta }_{t}$ coefficients make a jump at year 0 and become significantly greater than zero (*p* value < 0.05). After that the coefficients remain significant for the subsequent five to six years reaching a maximum in the fourth year (2012) since the onset of the crisis. After 2015 the estimated coefficients become not significant, and in 2019 the absolute value of the coefficients come back to the pre-crisis level.

The analysis in Fig. 3 confirms three relevant points. First, the crisis produced a significant increase in unemployment in Europe (hardly a surprise); second, the year 2009 is the year when the crisis first translated into a significant increase in unemployment; third that the classification proposed by Tapia Granados and Ionides (2017) is largely correct: both groups 2 and 3 show significantly higher unemployment rates compared to group 1 for at least five to six years after the onset of the crisis. Furthermore, group 3 countries present higher ${\delta }_{t}$ coefficients compared to group 2 countries for all years after the crisis.

## The effect of the Great Recession on life expectancy

**Fig. 4:**
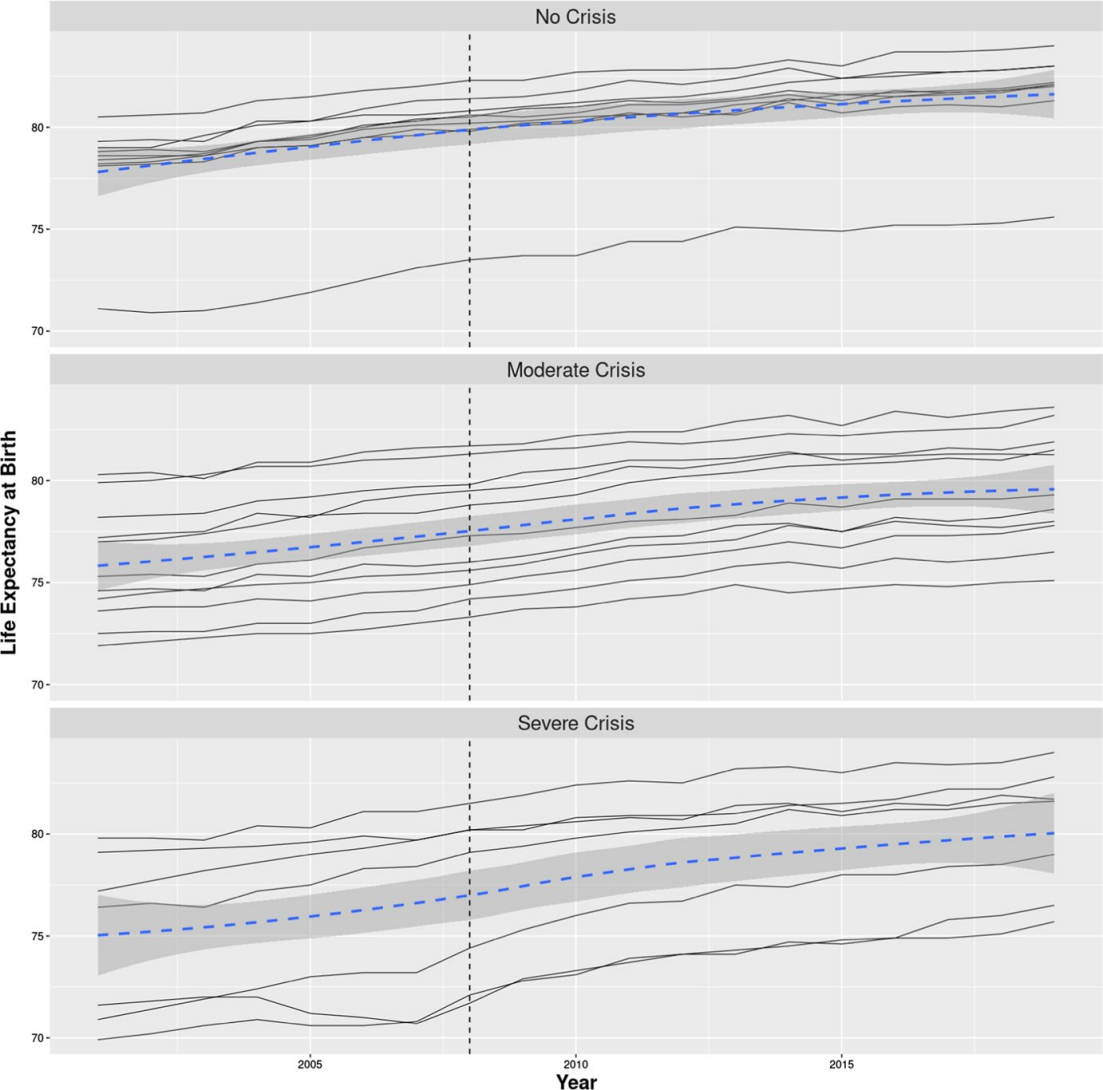
Evolution of life expectancy at birth in 27 European countries. *Note:* Countries have been classified into three main groups (no, moderate, and severe crisis) following the classification proposed by Tapia Granados and Ionides (2017)*.* The vertical dashed line indicates the last pre-crisis year, 2008. The blue dashed line has been estimated through local polynomial regression (LOESS), whereas the grey area represents the 95% confidence interval around this estimate. *Source*: Eurostat

If the Great Recession had randomly affected European countries, we could have considered it as a gigantic randomiszed experiment. In this case it would have sufficed to compare the mean life expectancy of the countries spared by the crisis (the control group) with that of the countries hit by the crisis (the treated group) to know the average causal effect of the crisis on life expectancy. However, crises do not hit at random, and this is the ultimate reason for employing methods such as panel data analysis or difference-in-differences to gauge their effects. The non-random nature of the Great Recession is clearly shown by Fig. 4 where we have represented the evolution of life expectancy over the period 2001–2019 for all 27 European countries analysed. As before, these countries have been classified into three main groups according to the severity of the crisis they experienced. Figure 4 shows that in the control group life expectancy in 2008 is not far from 80 years in all countries, Romania, with a life expectancy just above 73 years, is the only exception to this rule. The two remaining groups of countries are, in this respect, much more heterogeneous, with life expectancy spreading in 2008 from just above 70 years to just above 80 years. Figure 4 is thus telling us that the countries with higher life expectancy have a lower likelihood of being hit by the Great Recession. This fact may represent a potential problem for our analysis if it turns out that life expectancy increases more rapidly in countries with lower life expectancy, because this would represent a violation of the parallel trends assumption. For this reason, we need to check the estimates of Eq. (2) to exclude the possibility of this form of bias. As we discussed in “Data and methods” section this check will be performed through the estimate of Eq. (3).

Figure 4 also shows that life expectancy trajectories at country level follow approximately a linear trend. This pattern appears to be violated only in a few cases. In three countries that experienced a severe crisis, Estonia, Latvia and Lithuania, life expectancy appears to stall or even slightly decline in the period 2004–2007. The period 2004–2007, preceding the crisis, was in the Baltic States a period of strong economic growth. According to the estimates given by Tapia Granados and Ionides (2017) unemployment dropped by 5.3 percentage points in Estonia, by 3.9 percentage points in Latvia and by 7.0 percentage point in Lithuania. Consistently with the theory of pro-cyclical mortality, we observe in these countries, during the phase of economic boom, a stall in life expectancy. On top of that, in 2 years, 2003 and 2015, several countries show a temporary decline in life expectancy provoked, in both cases, by a heat wave and, in the case of 2015, also by an unusually severe flu epidemic (Ballester et al., 2019; Mølbak et al., 2015). Apart from these local departures from linearity, the evolution of life expectancy follows an almost parallel trend across European countries. The aim of this section is to assess whether the start of the Great Recession has modified these trends so that, after 2008, life expectancy shows a faster increase in the countries more affected by the crisis compared to those spared by it.

**Fig. 5:**
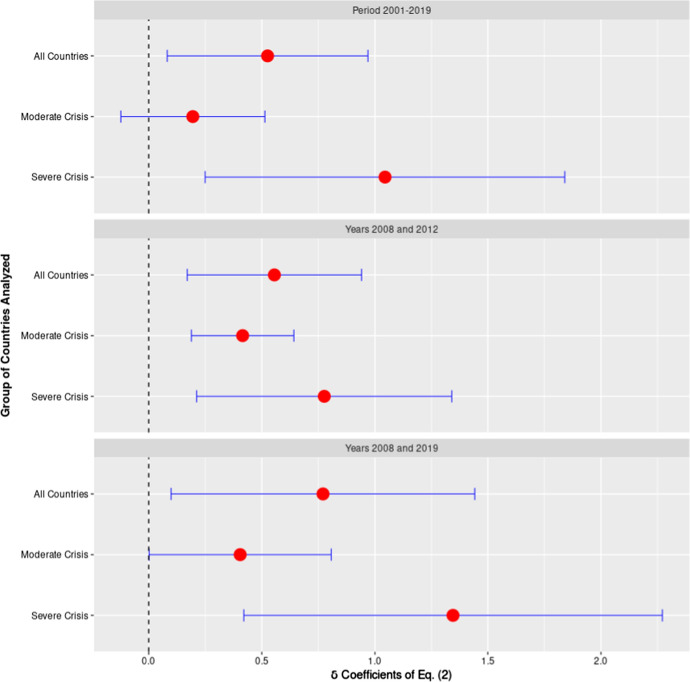
The Effect of the Crisis on Life Expectancy at Birth. *Note:* This figure represents the estimated $\delta$ coefficient of Eq. (2) along with their 95% confidence intervals. In the 9 estimates displayed by this figure the control group is always represented by the countries that suffered no crisis or a mild form of it. The treated group varies according to the kind of analysis performed: *All Countries* considers together as the treated group, group 2 and 3 countries; *Moderate Crisis* considers as treated only group 2 countries; *Severe Crisis* considers as treated only group 3 countries. The first panel of Fig. 5 show the analysis performed over the entire 2001–2019 period. In the second and third panels the analysis is restricted to, respectively, the years 2008 and 2012, and 2008 and 2019. *Source*: Authors’ computation on Eurostat data

In Fig. 5 we show 9 estimates of the $\delta$ coefficient of Eq. (2) along with their 95% confidence intervals. These analyses have been performed with different sets of countries and with different periods. In all analyses the control group is represented by the group 1 countries (no crisis). The treated group varies, instead, according to the kind of analysis performed: *All Countries* considers together as a treated group group 2 and 3 countries; *Moderate Crisis,* considers as treated only the group 2 countries (dropping from the analysis the group 3 countries); *Severe Crisis* considers as treated only group 3 countries (dropping from the analysis group 2 countries). The first panel of Fig. 5 shows the analysis performed over the entire period 2001–2019. In the second and third panels the analysis is restricted to, respectively, the years 2008 and 2012, and 2008 and 2019.

The first panel (Period 2001–2019) of Fig. 5 shows that when all countries that experienced a moderate or a severe crisis are considered together as a treated group the delta coefficient attains the value of 0.5 years (*p* value < 0.05) indicating that the crisis produced in the treated group an average gain in life expectancy (over the following 11 years) of about half a year compared to the control group. If we compute separately the delta coefficient for the group 2 and 3, we get an 0.2 years (*p* value > 0.05) gain in life expectancy for moderate crisis countries, whereas the gain for severe crisis countries is about 1 year (*p* value < 0.05). This is the first of our placebo tests, which indicates that the gain in life expectancy is larger in those countries where the crisis hit harder. All placebo tests that we performed confirm the goodness of the classification of the European countries adopted for this study and the overall soundness of the study design.

To assess the long-term effect of the crisis on life expectancy, we repeated the same analysis but selecting specific points in time: we first consider the years, 2008, the last pre-crisis year, and 2012, the year when the unemployment rate was at its maximum (see Fig. 3); then we considered the years, 2008 and 2019. In this way we performed two analyses that resemble very closely a simplified 2 × 2 DD design. The results of these analyses are very similar to that obtained considering the whole period 2001–2019. The results presented in the last panel (years 2008 and 2019) of Fig. 5 show that the countries that were more affected by the crisis are still associated with a gain in life expectancy in 2019. In fact, the estimated coefficients are generally larger in this last analysis than in the previous ones, suggesting that the beneficial (for life expectancy) effects of the crisis have probably cumulated over time. This is a surprising result if one considers that in 2019 unemployment rates have practically come back to their pre-crisis level (see “Unemployment changes during and after the Great Recession” section).

**Fig. 6:**
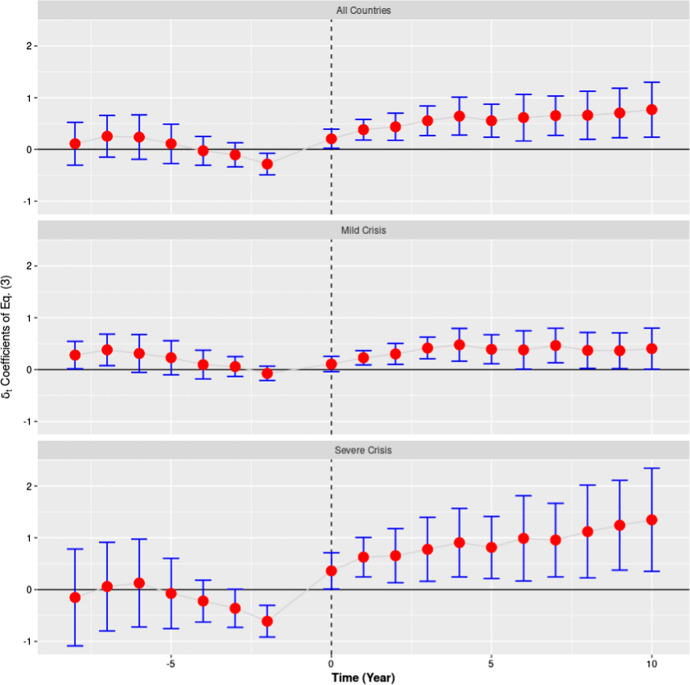
Robustness check on the effect of the crisis on life expectancy in three groups of European countries. *Note:* This figure represents the estimated $\delta_{t}$ coefficients of Eq. (3) along with their 95% confidence intervals. The dependent variable is, in this case, Life expectancy at birth. In the three analyses displayed by this figure (All Countries, Moderate Crisis and Severe Crisis) the control group is always represented by the countries that suffered no crisis or only a mild form of it. In the first panel (*All Countries*) the model has been estimated for all 27 countries considering together as a treated group the countries that suffered a moderate or a severe crisis. In the second panel (*Moderate Crisis*, 20 countries) the treated group is represented by the countries that went through a moderate crisis, whereas the countries that experienced a severe crisis were dropped from the analysis. In the third panel (*Severe Crisis*, 16 countries) the treated group is represented by the countries that went through a severe form of crisis, whereas the countries that experienced a moderate crisis were dropped from the data set. The vertical dashed lines indicate the first crisis year, 2009. *Source*: Authors’ computation on Eurostat data

This long-lasting effect is also confirmed by the estimation of the ${\delta }_{t}$ coefficients of Eq. (3) reproduced in Fig. 6. As in our previous analyses, these coefficients have been computed comparing the same control group (no Crisis) with three different treated groups: All Countries, Moderate Crisis and Severe Crisis. With the onset of the crisis, we observe in all three panels an increase in the value of the ${\delta }_{t}$ coefficients. In the group of countries which that experienced a severe crisis this increase continues until the end of our series reaching a value of about 1.5 years in 2019. In the countries that suffered, instead, a moderate crisis the increase stops around 2013, followed by a plateau at around 0.5 years. In both cases the ${\delta }_{t}$ coefficients prove to be statistically different from zero for the period 2009–2019.

Figure 6 also shows that the ${\delta }_{t}$ coefficients are, with few exceptions, not statistically different from zero before the crisis onset (for *t* < 0). Overall, 21 tests were performed on the pre-crisis period (2000–2007) to test the parallel trends assumption and only 4 of them turned out to be significantly different from 0 at a 5% significance level. Two checks failed in the period 2001–2002 in the “moderate crisis group”. These years are characteriszed by a short economic crisis that mostly affected the countries in the “moderate crisis group”. Two checks failed in the year 2007 both in the “Severe crisis” and the “All countries” groups. This was likely produced, as we have already seen, by the economic boom of the Baltic States. Notice that in both situations the behaviour of our series is consistent with the predictions of the procyclical theory of mortality: during the 2000–2001 crisis the countries in the “moderate crisis” show a gain in life expectancy, whereas during the boom of Baltic States life expectancy progressed at a slower pace. Figure 6 indicates that the hypothesis that the evolution of life expectancy is faster in the set of countries with lower life expectancy can be rejected for the countries and the epoch here analysed and that there are not systematic deviations from the parallel trend hypothesis.

It is possible that local deviations from the parallel trends hypothesis—that we have identified—could have biased in part the results presented in the first panel of Fig. 5 concerning the period 2001–2019. However, the two analyses concerning the years 2008, 2012 and 2008, 2019 are sheltered from these perturbations. Furthermore, since all different analyses performed (Figs. 5, 6) show very similar results, this indicates that our estimates are overall robust.

**Fig. 7:**
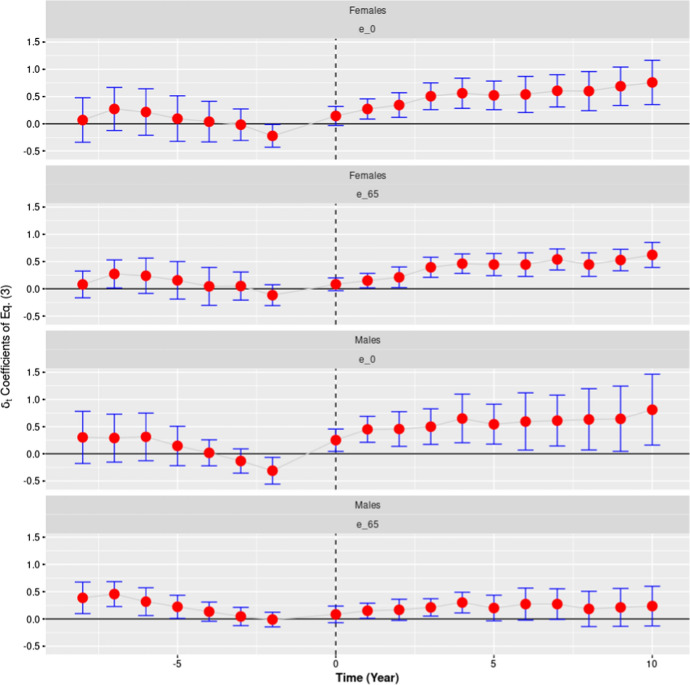
The Effect of the Crisis on Life Expectancy by Age and Sex. *Note:* This figure represents the estimated $\delta_{t}$ coefficients of Eq. (3) along with their 95% confidence intervals. “e_0” and “e_65” indicate respectively that life expectancy at birth and life expectancy at age 65 have been used as a dependent variable in Eq. (3). In all analyses displayed, the control group is represented by the countries that suffered no crisis or only a mild form of it. The treated group is represented, instead, by those countries which experienced a moderate or a severe crisis. The two top panels (*Females*—*e_0*, and *Females e_65*) show the results of the analyses carried out on females. The two bottom panels (*Males*—*e_0*, and *Males e_65*) show the results of the analyses carried out on males. The vertical dashed lines indicate the first post-crisis year, 2009. *Source*: Authors’ computation on Eurostat data

In our last analysis, displayed in Fig. 7, we have estimated the ${\delta }_{t}$ coefficients of Eq. (3) separately for males and females using, as a dependent variable, life expectancy at birth and at age 65. We used, as in our previous analysis, the group 1 countries as a control group, and as treated group the group 2 and 3 countries taken together (we did not perform a placebo test in this case). When the analysis is performed using life expectancy at birth no significant differences emerge between males and females: in both cases we observe an increase in the ${\delta }_{t}$ coefficients of the same magnitude over the entire period 2009–2019. Things change, however, when we consider life expectancy at age 65.

In this case, the crisis appears to have had a more important positive effect on females than on males. This result seems interesting to us for two reasons: First, it indicates that the beneficial effect of the crisis extends over a population (65+) which is comparatively more sheltered by the direct economic consequences of the crisis; second, it appears consistent with the analysis performed by Stevens et al. (2015) in the US context, suggesting that at least some of the causal mechanisms linking together the dynamics of unemployment and life expectancy may be similar in Europe and the US.

## Discussion

By employing a DD research design, we tried in this paper to look from a different perspective at the effects of the Great Recession on European life expectancy. Most of our results confirm what has already been found in previous research works: the rise in unemployment is associated with a gain in life expectancy; the magnitude of the gain reflects the severity of the crisis; for life expectancy at birth, males and females behave similarly, but when the analysis focuses on the population aged 65+, females appear to benefit more from the crisis than males.

Our results differ from previous research works in only one respect: the crisis had a long-lasting effect on life expectancy still detectable 11 years after its onset. Actually, life expectancy appears to have responded quickly to the rise in unemployment which occurred after 2008. Nevertheless, the gain in life expectancy accumulated over the years 2009–2012, when unemployment was on the rise, was preserved, and sometimes increased, in the subsequent period 2013–2019, when Europe saw a general reduction in the unemployment rate. The dynamics of life expectancy appears, thus, asymmetric: it responds quickly to an increase but not to a decrease in unemployment. The outbreak of the COVID-19 epidemics prevents us from knowing whether the effects of the crisis would have lasted after 2019. This notwithstanding, the relationship between mortality and the economic cycle does not appear, in the context analysed here analyzed, to be purely short-term.

Although contemporary economic crises have a cyclical component, it is possible that the Great Recession presents some unique features that cannot be generaliszed. The Great Recession is considered the end of a long phase that begins in the mid-1980s, the Great Moderation, characteriszed by a reduced volatility of business cycle fluctuation. The crises that took place during this phase, such as the 2000–2001 crisis, were comparatively mild and brief with respect to the Great Recession. We cannot exclude, therefore, that in the case of the Great Recession mechanisms different from those previously operating have been activated. So, even though unemployment levels have gradually returned to their pre-crisis levels in the aftermath of the crisis, other relevant economic indicators may have experienced long-lasting change.

Although we cannot exclude this possibility, we want to note here that the long-lasting effects on life expectancy produced by the Great Recession are, at least in principle, consistent with some of the explanations already advanced to justify the procyclical dynamics of mortality. If individuals during the slowdown of the economy tend to modify their lifestyle, quitting smoking, stopping drinking, doing more physical activity, going more frequently to the doctor etc. we can suppose that at least some of these healthier behaviors are preserved after the crisis, and perhaps for a long time after it.

The DD research design seems to us a well-matched method for analyszing the effect of the crisis on life expectancy, for three main reasons: (1) period life expectancy tends to evolve linearly following parallel trends in most European countries (see Fig. 4); (2) the Great Recession was largely an unforeseen event (Greenspan, 2013; Lin & Treichel, 2012); and (3) the onset of the crisis was almost simultaneous in the 27 European countries covered here (see Fig. 2). However, our analysis also identified several potential problems for the application of this method that should be borne in mind when interpreting our results.

One important problem is represented by the fact that in the Baltic States life expectancy deviates from a parallel trajectory in the years immediately before the crisis (see Fig. 4). Furthermore, the 2000–2001 crisis may have also induced some perturbations in the evolution of life expectancy during the period before the Great Recession. These are some of the reasons that led us to repeat our analysis focusing on specific points in time (Fig. 5).

The identification of the exact timing of the crisis may be considered somewhat arbitrary. For instance, Eurostat (2009), using monthly data from the European Labor Force Survey, identifies the average onset of the crisis in March 2008, when unemployment reached in Europe its minimum level. Using annual data, instead, the onset of the crisis moves somewhat forward to 2009 (see Fig. 2). Since our analyses uses annual data, we preferred to take 2009 as the start of the crisis.

To perform our analysis and the placebo tests, we classified the European countries into three groups according to the severity of the effects produced by the crisis. Of course, this is, to a certain extent, an arbitrary classification. It must be said, however, that this classification was proposed independently from this study, by different authors, employing different methodologies. It cannot be considered, therefore, as being intended to maximize the value of the $\delta$ coefficients in our equations.

Finally, the fact that the crisis had a positive effect on life expectancy should not distract from its negative effects. Several countries saw during the crisis, and after it, long-lasting stagnation, or an absolute contraction in *per capita* health-care expenditure. After 2011, in Greece, the funding of the health care system was so compromised and the economic effects of the crisis so severe that they were considered to be the “omens of a Greek tragedy” (Kentikelenis et al., 2011). Even if the impact of the crisis on Greek mortality turned out to be “more nuanced” than previously hypothesiszed (Filippidis et al., 2017), it is hard to believe that the reduction in the health-care funding did not entail negative effects on health and mortality. We also saw that there are few doubts that unemployment entails negative health outcomes on the population directly affected by it. The DD procedure is not able, at least in the implementation here presented, to disentangle these different effects: what is measured in our approach is simply the algebraic sum of the positive and the negative effects produced by the crisis. Indeed, the fact that the positive effects eventually prevail suggests that these effects may have been even stronger than our findings show.
